# The role of interim FDG PET-CT after induction chemotherapy as a predictor of concurrent chemoradiotherapy efficacy and prognosis for head and neck cancer

**DOI:** 10.1007/s00259-017-3836-8

**Published:** 2017-09-22

**Authors:** Ka-Rham Kim, Hyun-Jeong Shim, Jun-Eul Hwang, Sang-Hee Cho, Ik-Joo Chung, Ki Seong Park, Sae-Ryung Kang, Seong Young Kwon, Woong-Ki Chung, Woo Kyun Bae

**Affiliations:** 1 Department of Hemato-Oncology, Chonnam National University Medical School, Gwangju, Republic of Korea; 2 Department of Nuclear Medicine, Chonnam National University Medical School, Gwangju, Republic of Korea; 3 Department of Radiation-Oncology, Chonnam National University Medical School, Gwangju, Republic of Korea; 4 Division of Hematology-Oncology, Department of Internal Medicine, Chonnam National University Hwasun Hospital, 322, Seoyang-ro, Ilsim-ri, Hwasun-eup, Hwasun-gun, Jeollanam-do 58128 Republic of Korea

**Keywords:** Head and neck cancer, Induction chemotherapy, Concurrent chemoradiotherapy, PET-CT

## Abstract

**Purpose:**

Induction chemotherapy (ICT) with docetaxel, cisplatin, and 5-fluorouracil (TPF) followed by concurrent chemoradiotherapy (CCRT) has the advantages of organ preservation and systemic control in head and neck cancer (HNC). Early prediction of CCRT efficacy may help identify patients who will benefit more from surgery than from CCRT. We investigated the role of interim 18-fluoro-2-deoxy-glucose positron emission tomography computed tomography (FDG PET-CT) after ICT to predict the efficacy of CCRT and clinical outcomes.

**Methods:**

Tumor responses were retrospectively reviewed after CCRT based on the Response Evaluation Criteria in Solid Tumors. FDG PET-CT imaging was performed before and after three cycles of TPF. We examined the associations between the metabolic response (percentage decrease in the maximum standardized uptake value [SUVmax] and total metabolic tumor volume [MTV]) after ICT and complete response (CR) to CCRT, progression-free survival (PFS), and overall survival (OS).

**Results:**

We studied 43 HNC patients with a median follow-up of 32.7 months. Lymph node (LN) SUVmax and total MTV decreases from baseline after ICT were greater in patients with a CR to CCRT than in non-CR patients (LN SUVmax, 88.8% vs. 62.5%, respectively; total MTV, 99.7% vs. 89.9%, respectively). Decreases in total MTV ≥ 78% and LN SUVmax ≥73% after ICT predicted CR to CCRT and longer OS and PFS.

**Conclusions:**

Using interim FDG PET-CT to measure SUVmax and total MTV after three cycles of ICT may be a useful technique for identifying HNC patients who will benefit from CCRT and predicting survival outcomes.

**Electronic supplementary material:**

The online version of this article (10.1007/s00259-017-3836-8) contains supplementary material, which is available to authorized users.

## Introduction

Concurrent chemoradiotherapy (CCRT) is the standard treatment for nonsurgical management of locally advanced head and neck cancer (HNC) [1]. Although the role of induction chemotherapy (ICT) is controversial, interest in ICT followed by CCRT has been renewed in recent years. Advances in locoregional therapies, such as surgery, radiotherapy (RT) and CCRT, have been accompanied by an increase in distant metastases as a result of treatment failure, which has stimulated interest in systemic therapies such as ICT [2]. Previous studies have reported an association between ICT and fewer distant metastases [3] and a higher radiological complete response (CR) when ICT was administered before CCRT [4]; this suggests that ICT may promote organ preservation and help to avoid surgery, which is associated with high morbidity. Several studies have shown that response rates, disease-free survival, and overall survival (OS) were significantly improved in patients who received ICT with taxane, cisplatin, and 5-fluorouracil (5-FU; TPF) compared with those receiving cisplatin and 5-FU alone [5–8]. However, TPF has not been shown to have a clear advantage over CCRT alone in terms of OS [9, 10].

Recently, early responses to chemotherapy were reported to predict survival outcomes in metastatic colorectal cancer [11]. Furthermore, the HNC response to ICT has been shown to predict the response to subsequent RT and disease control [12, 13]. Given that a good response to ICT is associated with a better outcome with combined chemotherapy and RT, it is possible that the response to ICT could be used to identify patients who will benefit more from further treatment with CCRT than from surgery. However, there is little evidence to suggest that the response to triple induction therapy is predictive of the response to subsequent CCRT.

18-fluoro-2-deoxy-glucose positron emission tomography computed tomography (FDG PET-CT) can be used to assess the response to therapy, as well as for diagnosis and staging workup. Several studies have used the metabolic response to FDG PET-CT as a prognostic marker of treatment outcome in HNC. Metabolic tumor volume (MTV) and the maximum standardized uptake value (SUVmax) before CCRT are associated with OS, local control [14, 15], and response to CCRT [16]. Additionally, percentage changes in SUVmax and MTV during ICT are associated with local control rate, progression-free survival (PFS), and OS [17–19]. Although several studies have investigated metabolic responses in relation to OS, few have examined the direct association between changes in SUVmax and MTV and the response to CCRT. Changes in SUVmax after doublet chemotherapy with S-1 and cisplatin have been shown to predict CR after CCRT [20]. Further larger studies using triple ICT are needed to support the potential clinical utility of interim FDG PET-CT to predict outcome and response to CCRT.

We investigated the role of interim FDG PET-CT after triple ICT as a predictor of the efficacy of CCRT in HNC.

## Materials and methods

### Patients and tumor assessment

We retrospectively reviewed the medical records and imaging data of HNC patients who underwent ICT followed by CCRT at Chonnam National University Hwasun Hospital between September 2008 and August 2014.

Eligible patients had histologically proven squamous carcinoma and nasopharyngeal carcinoma of the head and neck, were initially diagnosed as stage II to IVB according to the American Joint Committee on Cancer criteria, and were not suitable for surgery. FDG PET-CT imaging was performed before and 1 day after three cycles of ICT to assess the metabolic response. Tumor responses were evaluated using the modified Response Evaluation Criteria in Solid Tumors, metabolic response in FDG PET-CT and laryngoscopic examination after three cycles of ICT and 8 weeks after the completion of CCRT. Patients with another primary malignancy, and those who did not receive planned chemotherapy due to severe concomitant illness, were excluded from the study. In total, 47 patients with HNC received ICT assessed by interim FDG PET-CT. However, three patients received RT alone due to their poor general condition and one patient received five cycles of ICT; thus, the data of 43 patients were collected until patient death or April 2016.

Our study was approved by the Institutional Review Board of Chonnam National University Hwasun Hospital.

### Treatment

The patients received docetaxel, cisplatin, and 5-FU (TPF) ICT consisting of docetaxel (70 mg/m^2^) and cisplatin (75 mg/m^2^) on day 1 and 5-FU (1000 mg/m^2^) on days 1–4 of every 3-week cycle. All participants received three cycles of TPF.

CCRT was started within 5 weeks of the last ICT cycle. RT was administered 5 days/week with daily fractions of 1.8 or 2.0 Gy. The median radiation dose was 66.0 Gy (range: 43.2–72.0 Gy). We treated 32 (74.4%) patients with intensity-modulated radiation therapy and the remaining 11 (25.6%) were treated with three-dimensional conformal RT. The concurrent chemotherapy regimen was one dose of cisplatin (75–100 mg/m^2^) every 3 weeks.

### Imaging acquisition and measurement of metabolic parameters

FDG PET-CT was performed using the Discovery ST PET-CT system (GE Medical Systems, Milwaukee, WI, USA). Briefly, all patients fasted for 6 h before receiving an intravenous injection of FDG. A low-dose CT scan was performed for attenuation correction 50 min after the FDG injection (5.55 MBq/kg body weight). Then PET images were acquired for 150 s per bed position. Data were reconstructed using ordered subset expectation maximization reconstruction (128 × 128 matrix, 3.27-mm slice thickness; subset: 21, iterations: 2). The degree of FDG uptake was assessed using a semiquantitative technique in which a volumetric region of interest was placed over the FDG-avid lesions, and the highest value was selected (SUVmax). The MTV was measured by applying a fixed SUV threshold of 2.5 as the lowest limit of the segmentation criteria. MTV was measured in the primary site and metastatic lymph nodes (LNs), and the total MTV was defined as the sum of the primary and nodal MTVs. The metabolic parameters were measured on an Advantage Workstation (GE Healthcare, Milwaukee, WI, USA) using PET volume computer-assisted reading software (ver. 1.0).

### Statistical analysis

Continuous variables were expressed as means ± standard deviation. Chi-square or Fisher’s exact tests were used to compare categorical variables, and Student’s *t*-tests were used to compare continuous variables. Kaplan-Meier analysis was performed to assess cumulative survival and time to death or progression, and the log-rank test was used to assess the correlations between end points (CR, PFS and OS) and decreases in SUVmax or MTV. Multivariate analyses were performed using a Cox proportional hazards model and logistic regression analysis to identify independent prognostic variables (forward selection). A one-sided *p*-value of 0.05 was considered to indicate statistical significance. All statistical tests were performed using SPSS for Windows software (ver. 21.0; IBM Corp., Armonk, NY, USA).

## Results

### Patient baseline characteristics

The baseline characteristics of the patients are shown in Table 1. The study included 43 patients, of whom 38 (88.4%) were male and 5 (11.6%) were females. The median patient age was 61 years, and 20 (46.5%) patients were older than 65 years. The most common primary tumor sites were the hypopharynx (*n* = 16, 37.2%) and nasopharynx (*n* = 15, 34.9%). A total of 17 (39.5%) patients had stage IVA disease and 10 (23.3%) had stage IVB stage.

**Table 1:** Patient baseline characteristics

	Total, n (%) (*n* = 43)
Age (y, median)	61 ± 9.9
Sex (male)	38 (88.4)
ECOG PS	
0	16 (37.2)
1	24 (55.8)
2	3 (7.0)
Primary tumor sites	
Hypopharynx	16 (37.2)
Nasopharynx	15 (34.9)
Oropharynx	8 (18.6)
Oral cavity	2 (4.7)
Larynx	1 (2.3)
Paranasal sinus	1 (2.3)
Stage of primary tumor	
T1	6 (14.0)
T2	18 (41.8)
T3	5 (11.6)
T4	14 (32.6)
Nodal stage	
N0	2 (4.7%)
N1	15 (34.9%)
N2	23 (53.4%)
N3	3 (7.0%)
Overall stage of disease	
II	3 (7.0)
III	13 (30.2)
IVA	17 (39.5)
IVB	10 (23.3)

*ECOG PS* Eastern Cooperative Oncology Group Performance Status

### Treatment outcomes

Following ICT, 6 (14.0%) patients achieved CR and 36 (83.7%) achieved a partial response (PR). One (2.3%) patient showed progression after ICT and salvage surgery was recommended; however, the patient refused surgery and subsequently received CCRT.

After CCRT, 26 (60.4%), 13 (30.2%), 2 (4.7%), and 2 (4.7%) patients demonstrated CR, PR, stable disease, and progressive disease, respectively. Of the 26 patients who achieved CR, cancer recurred in 7 and 5 patients died of disease progression. Of the 17 non-CR patients, 8 died of disease progression. Thus, at last follow up, 13 (30.2%) patients had died. The median follow-up duration was 32.7 months (range: 7.2–88.3 months). The 2-year OS and PFS rates were 72.7% and 61.1%, respectively. The median OS and PFS were not reached.

### Association between decreases in SUVmax and total MTV after ICT and CR to CCRT

**Fig. 1:**
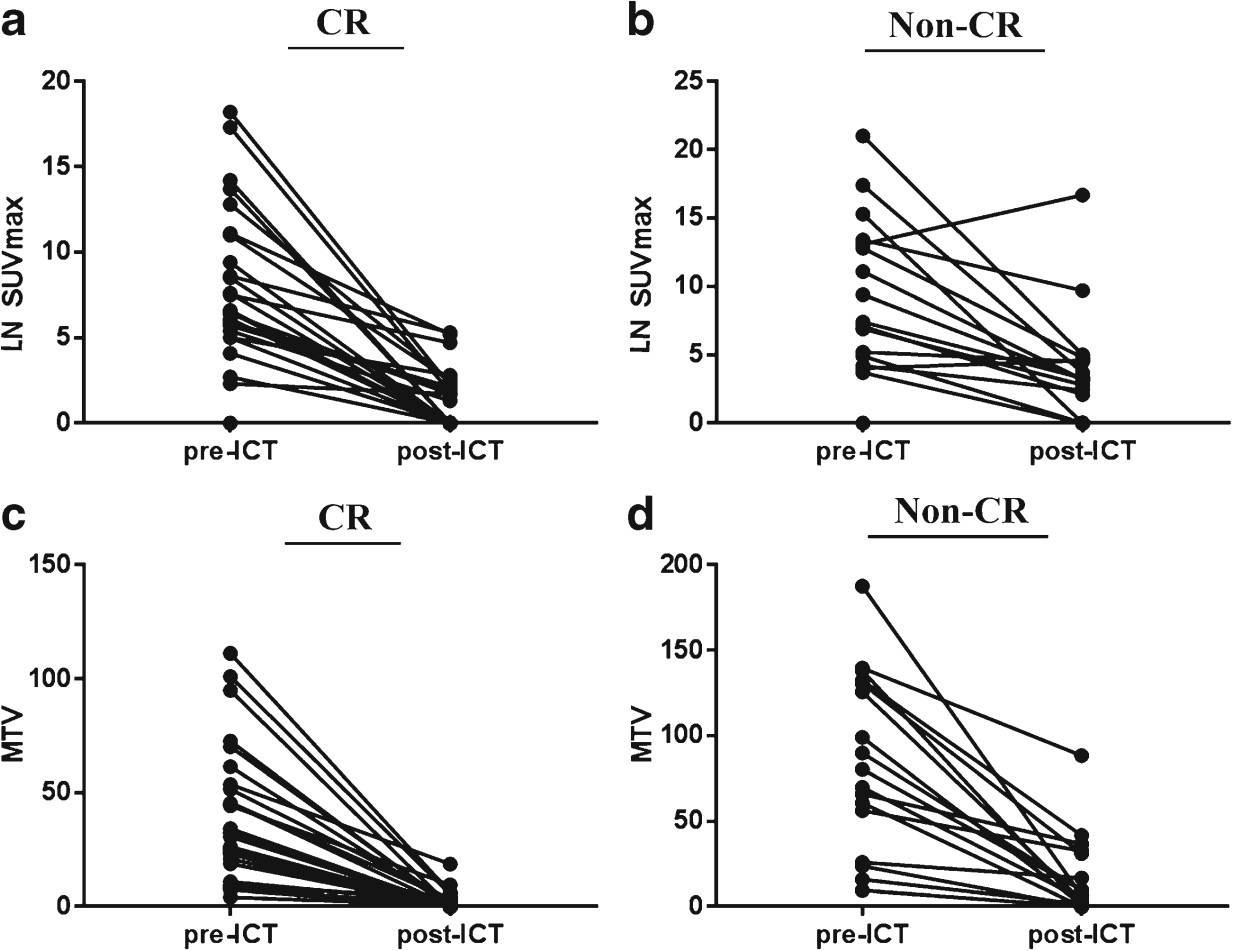
Changes in the maximum standardized uptake value (SUVmax) in the lymph node (LN) and total metabolic tumor volume (MTV) before and after induction chemotherapy (ICT). **a** LN SUVmax in patients who achieved complete response to ICT. **b** LN SUVmax in patients who did not achieve complete response to ICT. **c** Total MTV in patients who achieved complete response to ICT. **d** Total MTV in patients who did not achieve complete response to ICT

Baseline SUVmax (SUVmax0) and SUVmax after ICT (SUVmax1) of the primary and LN lesions are shown in Table 2. At baseline, the median LN SUVmax was 7.1 (range: 0.0–21.0), and after three cycles of ICT the median LN SUVmax decreased to 2.0 (range: 0.0–16.7). The median percentage decrease in LN SUVmax was 76.0% (range: 27.5–100.0%). Patients who achieved CR after CCRT showed a greater decrease in LN SUVmax than non-CR patients (88.8 vs. 62.5%, *p* = 0.009; Table 2, Fig. 1a, b).

**Table 2:** Changes in SUVmax according to response to concurrent chemoradiotherapy

	Total	Non-CR (*n* = 17)	CR (*n* = 26)	*p*-value^a^
Primary lesion				
SUVmax0, median [range]	10.8 [0.0–26.2]	16.0 [3.5–21.0]	8.85 [0.0–26.2]	
SUVmax1, median [range]	2.6 [0.0–16.0]	3.6 [0.0–16.0]	2.2 [0.0–7.8]	
SUVmax decrease in percentage (%), median [range]	76.3 [−2.2–100.0]	67.7 [−2.2–100.0]	79.9 [0.0–100.0]	0.283
LN lesion				
SUVmax0, median [range]	7.1 [0.0–21.0]	7.4 [0.0–21.0]	6.6 [0.0–18.2]	
SUVmax1, median [range]	2.0 [0.0–16.7]	3.2 [0.0–16.7]	0.7 [0.0–5.3]	
SUVmax decrease in percentage (%), median [range]	76.0 [−27.5–100.0]	62.5 [−27.5–100.0]	88.8 [0.0–100.0]	0.018

*SUVmax* maximum standardized uptake value, *CR* complete response, *LN* lymph node

^a^Student’s *t*-test

The median total MTV at baseline (MTV0) was 45.7 (range: 4.1–187.6), and the median total MTV after three cycles of ICT (MTV1) was 1.0 (range: 0.0–88.4). The median decrease in total MTV was 99.7% in patients who achieved CR after CCRT and 89.9% in non-CR patients (*p* = 0.020; Table 3, Fig. 1c, d).

**Table 3:** Changes in MTV according to complete response to concurrent chemoradiotherapy

	Total	Non–CR (n = 17)	CR (n = 26)	*p*-value^a^
Primary lesion				
MTV0, median [range]	18.6 [0.0–159.0]	34.2 [2.2–159.0]	17.3 [0.0–99.8]	
MTV1, median [range]	0.3 [0.0–88.4]	4.5 [0.0–88.4]	0.0 [0.0–12.8]	
MTV decrease in percentage (%), median, [range]	98.8 [0.0–100.0]	96.5 [28.1–100.0]	99.7 [0.0–100.0]	0.373
LN				
MTV0, median [range]	11.3 [0.0–119.0]	21.5 [0.0–119.0]	9.4 [0.0–78.2]	
MTV1, median [range]	0.0 [0.0–32.6]	0.1 [0.0–32.6]	0.0 [0.0–5.9]	
MTV decrease in percentage (%), median [range]	100.0 [0.0–100.0]	97.3 [0.0–100.0]	100.0 [0.0–100.0]	0.562
Total				
MTV0, median [range]	45.7 [4.1–187.6]	80.4 [9.6–187.6]	33.1 [4.1–111.2]	
MTV1, median [range]	1.0 [0.0–88.4]	6.6 [0.0–88.4]	0.1 [0.0–18.7]	
MTV decrease in percentage (%), median [range]	97.2 [35.5–100.0]	89.9 [35.5–100.0]	99.7 [58.0–100.0]	0.040

*MTV*, metabolic tumor volume, *CR* complete response, *LN* lymph node

^a^Student’s *t*-test

Our examination of proportional decrease, in LN SUVmax and total MTV, to identify the optimal cutoff value, revealed several rates of LN SUVmax and total MTV that predicted CR to CCRT, PFS, and OS. A 73% decrease in LN SUVmax was the strongest predictor of CR to CCRT (*p* = 0.007), PFS (*p* = 0.011) and OS (*p* = 0.003). For total MTV, a cutoff of a 78% decrease predicted CR after CCRT (*p* = 0.021), PFS (*p <* 0.001), and OS (*p <* 0.001; Supplementary Table 1).

We performed a logistic regression analysis including age, sex, performance status (PS), stage, tumor site, ≥ 73% decrease in LN SUVmax, and ≥78% decrease in total MTV to identify prognostic markers for CR after CCRT. The univariate and multivariate analyses revealed that a ≥ 73% decrease in LN SUVmax and total MTV decrease ≥78% were significantly associated with CR after CCRT (Table 4).

**Table 4:** Univariate and multivariate analyses of complete response to concurrent chemoradiotherapy

Variables	HR	95% CI	*p*-value
Univariate analysis			
SUVmax of LN decrease ≥73%	5.4	1.4–20.5	0.013
Total MTV decrease ≥78%	6.5	1.1–37.7	0.036
Multivariate analysis			
SUVmax of LN decrease ≥73%	10.2	2.2–46.1	0.003
Total MTV decrease ≥78%	6.5	1.1–37.7	0.036

*SUVmax* maximum standardized uptake value, *MTV* metabolic tumor volume, *HR* hazard ratio, *CI* confidence interval

### Decreases in LN SUVmax and total MTV as prognostic markers

**Fig. 2:**
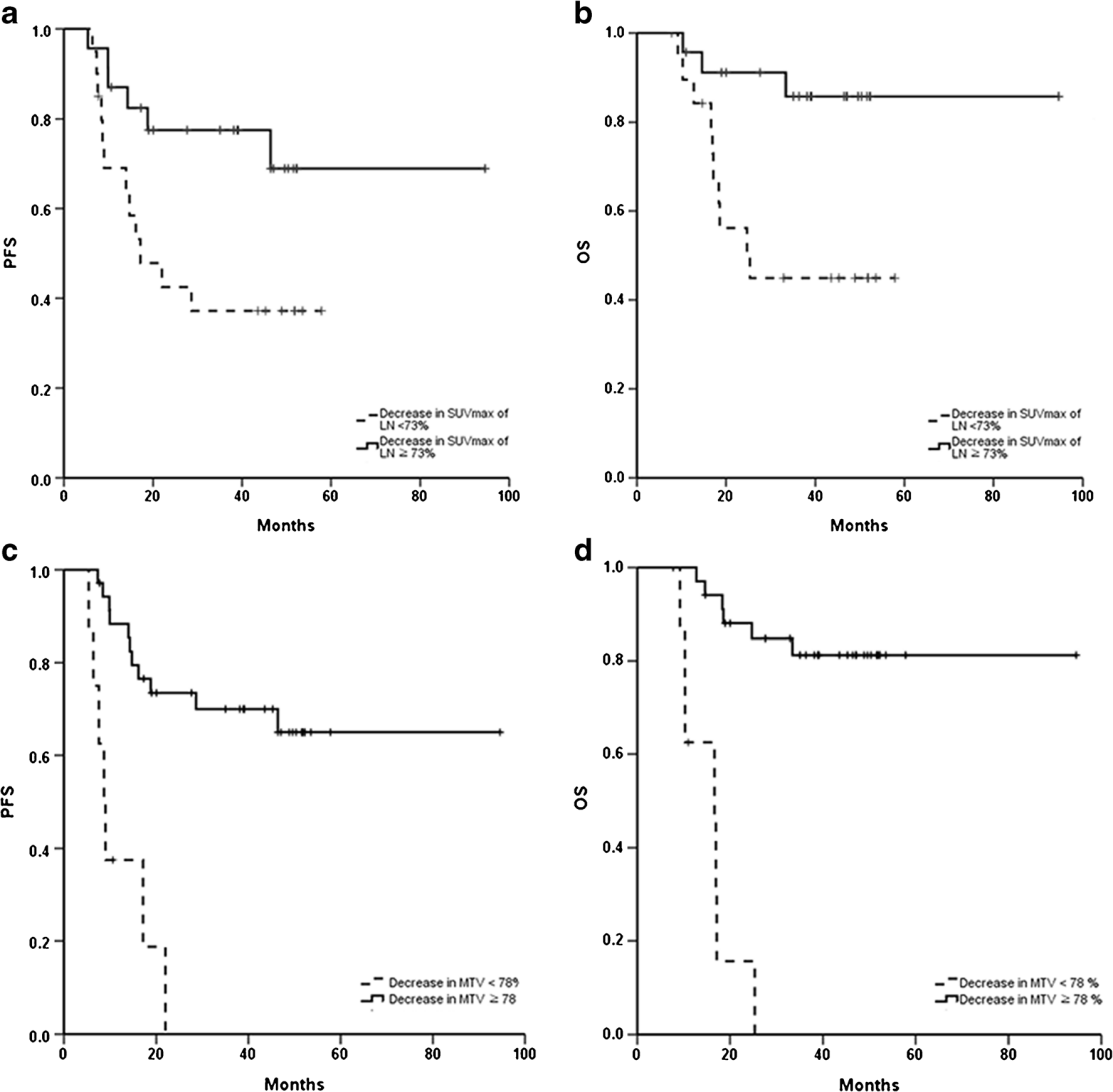
Progression-free survival (PFS) and overall survival (OS) according to the decrease in the LN SUVmax and total MTV (N = 43). **a** PFS according to <73% and ≥73% decrease in LN SUVmax. **b** OS according to <73% and ≥73% decrease in LN SUVmax. **c** PFS according to <78% and ≥78% decrease in total MTV. D) OS according to <78% and ≥78% decrease in total MTV

In terms of the LN SUVmax response, the median PFS in the poor metabolic response group (LN SUVmax decrease <73%) was 17.1 months, whereas the median PFS in the good response group (LN SUVmax decrease ≥73%) was not reached (hazard ratio [HR] = 5.3, *p* = 0.011). Furthermore, the poor metabolic response group had a shorter OS than the good response group (HR = 7.7, *p* = 0.003; Fig. 2a, b).

When patients were grouped according to total MTV response, the median PFS was 8.7 months in the poor metabolic response group (total MTV decrease <78%) but median PFS in the good response group was not reached (total MTV decrease ≥78%; HR = 20.4, *p* < 0.001). Similarly, the median OS was 16.6 months in the poor metabolic response group but median OS was not reached in the good response group (HR = 35.1, *p* < 0.001; Fig. 2c, d).

Additionally, we performed a Cox proportional hazard regression analysis to assess the utility of age, sex, PS, stage, tumor site, LN SUVmax decrease ≥73% and total MTV decrease ≥78% as prognostic markers for PFS and OS. The univariate analysis revealed that a ≥ 73% decrease in LN SUVmax and total MTV decrease ≥78% were associated with longer PFS and OS. The multivariate analysis revealed that a total MTV decrease ≥78% (HR = 7.5; 95% CI = 2.7–20.9; *p <* 0.001) and an LN SUVmax decrease ≥73% (HR = 3.0; 95% CI = 1.1–8.0; *p =* 0.014) were prognostic markers for PFS. Moreover, a ≥ 78% decrease in total MTV (HR = 17.0; 95% CI = 5.0–57.4; *p <* 0.001) and ≥73% decrease in LN SUVmax (HR = 5.2; 95% CI = 1.4–19.0; *p =* 0.003) were significantly associated with OS (Table 5).

**Table 5:** Univariate and multivariate analyses of progression-free survival and overall survival

Univariate analysis					
	No. of patients	Median PFS (months, 95% CI)	*p*-value	Median OS (months, 95% CI)	*p*-value
LN SUVmax decrease					
< 73%	20	17.1 (6.8–27.4)	0.021	25.4 (11.5–39.3)	0.006
≥ 73%	23	NA		NA	
Total MTV decrease					
< 78%	8	8.7 (6.7–10.7)	<0.001	16.6 (8.9–24.4)	<0.001
≥ 78%	35	NA		NA	
Multivariate analysis					
		HR	95% CI	*p*-value	
PFS					
Total MTV decrease ≥78%		7.5	2.7–20.9	<0.001	
LN SUVmax decrease ≥73%		3.0	1.1–8.0	0.028	
OS					
Total MTV decrease ≥78%		17.0	5.0–57.4	<0.001	
LN SUVmax decrease ≥73%		5.2	1.4–19.0	0.006	

*SUVmax* maximum standardized uptake value, *MTV* metabolic tumor volume, *PFS* progression-free survival, *OS* overall survival, *HR* hazard ratio, *CI* confidence interval

## Discussion

Our study is the first to investigate the role of interim FDG PET-CT after ICT with TPF as a predictor of the efficacy of CCRT for HNC.

ICT before RT or CCRT is a treatment option for advanced HNC, particularly for tumors in the oropharynx, hypopharynx, and larynx, where organ preservation is an important therapeutic consideration [1]. Triple therapy with TPF is the standard ICT regimen [5–8]. Salvage surgery is recommended for patients who do not achieve CR after CCRT; however, the complication rate of salvage surgery after CCRT is higher than that of primary surgery, possibly because poor quality tissue contributes to less than optimal wound healing and postoperative function [21]. Thus, early prediction of the response to CCRT would allow clinicians to consider surgery following ICT for patients predicted to have a poor response to CCRT. Furthermore, although the ICT response rate is generally high, clinical outcomes are mixed and CCRT after ICT can cause severe toxicity [22].

We found that interim FDG PET-CT after ICT could be used to assign patients to treatment and predict the outcomes of CCRT. Patients showing deterioration after ICT might show decreased CR with subsequent CCRT. FDG PET-CT enables clinicians to evaluate the metabolic response to therapy and identify possible biomarkers of outcome and survival. Studies comparing FDG PET-CT with CT have demonstrated higher efficacy of FDG PET-CT in patients with advanced HNC [23, 24]. Furthermore, FDG PET-CT after ICT has been shown to be a better predictor of outcome than the endoscopic response or magnetic resonance imaging results in patients with HNC [25, 26].

A decrease in FDG uptake after therapy has been associated with prognosis and CR in other malignancies [27, 28]. In HNC, a significant decrease in SUVmax after one cycle of ICT indicates a better prognosis and local control [17, 29]. Changes in SUVmax after two or three cycles of ICT with S-1 and cisplatin are associated with CR after CCRT, PFS, and OS [20]. However, because TPF ICT administered in three cycles is the treatment of choice, it would be premature to evaluate the response to ICT after the first cycle, and few studies have investigated the role of interim FDG PET-CT in triple ICT for HNC. All of our patients received the standard triple regimen and completed three cycles of ICT. Moreover, we investigated the associations of MTV and CR with survival outcomes.

We found that patients who achieved CR after CCRT showed greater decreases in LN SUVmax (88.8% vs. 62.5%, respectively; *p* = 0.009) and total MTV (99.7% vs. 89.9%, respectively; *p* = 0.020) after ICT than did non-CR patients. Moreover, multiple regression analysis revealed that a ≥ 73% decrease in LN SUVmax and ≥78% decrease in total MTV predicted CR after CCRT. Thus, we cautiously suggest that interim FDG PET-CT after ICT can be used to assign patients to treatment including CCRT and surgery.

Notably, a decrease in the LN SUVmax, and not in the primary site, was a predictive factor. The primary lesion may involve more inflammation and physiological uptakes than the LN after ICT, so a decrease in the SUVmax of the primary lesion could be underestimated. There was no difference in the SUVmax of the primary lesion after induction chemotherapy in the non-CR and CR groups (3.6 vs. 2.2, respectively, Table 2), so it could not be a predictive factor. Moreover, the LN was usually smaller than the primary lesion, and the decrease in the LN SUVmax could be overestimated due to partial volume effects.

We examined the association of changes in LN SUVmax and total MTV after ICT with survival outcomes. Several previous studies have shown that SUVmax and MTV before CCRT, and changes in SUVmax and MTV after ICT, are associated with survival [14, 15, 17, 18]. Our multivariate analysis revealed that changes in total MTV and LN SUVmax were associated with PFS and OS. However, changes in total MTV were more strongly associated with survival than were changes in SUVmax. This difference may be explained by the fact that MTV is a volumetric and metabolic biomarker of the tumor; thus, unlike SUVmax, MTV can quantify the overall tumor burden.

Our study had several limitations. First, it was a retrospective analysis of a relatively small number of patients. First, this study was retrospective. The PET scan time from our protocol was not sufficient to reproducibly evaluate the FDG uptake in tumors. A prospective study is therefore necessary to measure metabolic parameters within a consistent range and compare them before and after therapy. Second, patients in this study were heterogeneous. We used the stage and cancer site for parameters to predict outcomes, but they were not significant. When we divided the patients by cancer sites, nasopharynx (*n* = 15) vs. other sites (*n* = 28), there was no significant marker for predicting outcomes in the nasopharyngeal cancer group. But in the other cancer site group, the LN SUVmax decrease and total MTV decrease remained as valid parameters for predicting outcomes (Supplementary Table 2). Because these subgroups were too small to be reliable, further studies involving more patients are necessary. Third, our cutoff values were based on data from a single center; thus, further studies are needed to validate our findings. Nevertheless, we found marked differences in clinical outcomes when patients were classified as good or poor responders according to cutoff values, and our findings were consistent with those of a previous study [20]. Fourth, suspected residual lesions were confirmed by pathological biopsy, and the responses were assessed by radiological and laryngoscopic examination. Moreover, we found differences in incidence of chemotherapy during CCRT, and method of radiation among patients. However, we found no differences in CR, PFS, or OS according to the type of chemotherapy or method of radiation (data not shown).

Our findings suggest that interim FDG PET-CT after three cycles of triplet ICT may predict the efficacy of CCRT in patients with HNC, and we cautiously suggest that metabolic parameters measured by FDG PET-CT after ICT can be used to further assign patients to treatment and predict the prognosis after CCRT. However, future prospective studies with large sample sizes are needed to validate our findings.

## Electronic supplementary material

ESM 1 (DOC 92 kb)

## References

[1] Pfister DG, Spencer S, Brizel DM, Burtness B, Busse PM, Caudell JJ (2015). Head and neck cancers, version 1.2015. J Natl Compr Cancer Netw.

[2] Argiris A, Haraf DJ, Kies MS, Vokes EE (2003). Intensive concurrent chemoradiotherapy for head and neck cancer with 5-fluorouracil- and hydroxyurea-based regimens: reversing a pattern of failure. Oncologist.

[3] Lefebvre JL, Chevalier D, Luboinski B, Kirkpatrick A, Collette L, Sahmoud T (1996). Larynx preservation in pyriform sinus cancer: preliminary results of a European Organization for Research and Treatment of cancer phase III trial. EORTC head and neck cancer cooperative group. J Natl Cancer Inst.

[4] Paccagnella A, Ghi MG, Loreggian L, Buffoli A, Koussis H, Mione CA (2010). Concomitant chemoradiotherapy versus induction docetaxel, cisplatin and 5-fluorouracil (TPF) followed by concomitant chemoradiotherapy in locally advanced head and neck cancer: a phase II randomized study. Ann Oncol.

[5] Hitt R, Lopez-Pousa A, Martinez-Trufero J, Escrig V, Carles J, Rizo A (2005). Phase III study comparing cisplatin plus fluorouracil to paclitaxel, cisplatin, and fluorouracil induction chemotherapy followed by chemoradiotherapy in locally advanced head and neck cancer. J Clin Oncol.

[6] Posner MR, Hershock DM, Blajman CR, Mickiewicz E, Winquist E, Gorbounova V (2007). Cisplatin and fluorouracil alone or with docetaxel in head and neck cancer. N Engl J Med.

[7] Lorch JH, Goloubeva O, Haddad RI, Cullen K, Sarlis N, Tishler R (2011). Induction chemotherapy with cisplatin and fluorouracil alone or in combination with docetaxel in locally advanced squamous cell cancer of the head and neck: long-term results of the TAX 324 randomised phase 3 trial. Lancet Oncol..

[8] Vermorken JB, Remenar E, van Herpen C, Gorlia T, Mesia R, Degardin M (2007). Cisplatin, fluorouracil, and docetaxel in unresectable head and neck cancer. N Engl J Med.

[9] Haddad R, O’Neill A, Rabinowits G, Tishler R, Khuri F, Adkins D (2013). Induction chemotherapy followed by concurrent chemoradiotherapy (sequential chemoradiotherapy) versus concurrent chemoradiotherapy alone in locally advanced head and neck cancer (PARADIGM): a randomised phase 3 trial. Lancet Oncol.

[10] Cohen EE, Karrison T, Kocherginsky M, Huang CH, Agulnik M, Mittal BB et al. DeCIDE: a phase III randomized trial of docetaxel (D), cisplatin (P), 5-fluorouracil (F)(TPF) induction chemotherapy (IC) in patients with N2/N3 locally advanced squamous cell carcinoma of the head and neck (SCCHN). ASCO Annual Meeting Proceedings; 2012.

[11] Cremolini C, Loupakis F, Antoniotti C, Lonardi S, Masi G, Salvatore L (2015). Early tumor shrinkage and depth of response predict long-term outcome in metastatic colorectal cancer patients treated with first-line chemotherapy plus bevacizumab: results from phase III TRIBE trial by the Gruppo Oncologico del Nord Ovest. Ann Oncol.

[12] Urba S, Wolf G, Eisbruch A, Worden F, Lee J, Bradford C (2006). Single-cycle induction chemotherapy selects patients with advanced laryngeal cancer for combined chemoradiation: a new treatment paradigm. J Clin Oncol.

[13] Department of Veterans Affairs Laryngeal Cancer Study Group (1991). Induction chemotherapy plus radiation compared with surgery plus radiation in patients with advanced laryngeal cancer. N Engl J Med.

[14] Akagunduz OO, Savas R, Yalman D, Kocacelebi K, Esassolak M (2015). Can adaptive threshold-based metabolic tumor volume (MTV) and lean body mass corrected standard uptake value (SUL) predict prognosis in head and neck cancer patients treated with definitive radiotherapy/chemoradiotherapy?. Nucl Med Biol.

[15] Schwartz DL, Harris J, Yao M, Rosenthal DI, Opanowski A, Levering A (2015). Metabolic tumor volume as a prognostic imaging-based biomarker for head-and-neck cancer: pilot results from radiation therapy oncology group protocol 0522. Int J Radiat Oncol Biol Phys.

[16] Su M, Zhao L, Wei H, Lin R, Zhang X, Zou C (2015). 18F-fluorodeoxyglucose positron emission tomography for predicting tumor response to radiochemotherapy in nasopharyngeal carcinoma. Strahlenther Onkol.

[17] Kikuchi M, Nakamoto Y, Shinohara S, Fujiwara K, Yamazaki H, Kanazawa Y (2013). Early evaluation of neoadjuvant chemotherapy response using FDG-PET/CT predicts survival prognosis in patients with head and neck squamous cell carcinoma. Int J Clin Oncol.

[18] Yen RF, Chen TH, Ting LL, Tzen KY, Pan MH, Hong RL (2005). Early restaging whole-body (18)F-FDG PET during induction chemotherapy predicts clinical outcome in patients with locoregionally advanced nasopharyngeal carcinoma. Eur J Nucl Med Mol Imaging.

[19] Abgral R, Le Roux PY, Keromnes N, Rousset J, Valette G, Gouders D (2012). Early prediction of survival following induction chemotherapy with DCF (docetaxel, cisplatin, 5-fluorouracil) using FDG PET/CT imaging in patients with locally advanced head and neck squamous cell carcinoma. Eur J Nucl Med Mol Imaging.

[20] Yoon DH, Cho Y, Kim SY, Nam SY, Choi SH, Roh JL (2011). Usefulness of interim FDG-PET after induction chemotherapy in patients with locally advanced squamous cell carcinoma of the head and neck receiving sequential induction chemotherapy followed by concurrent chemoradiotherapy. Int J Radiat Oncol Biol Phys.

[21] Lee SC, Shores CG, Weissler MC (2008). Salvage surgery after failed primary concomitant chemoradiation. Curr Opin Otolaryngol Head Neck Surg.

[22] Ko EC, Genden EM, Misiukiewicz K, Som PM, Kostakoglu L, Chen CT (2012). Toxicity profile and clinical outcomes in locally advanced head and neck cancer patients treated with induction chemotherapy prior to concurrent chemoradiation. Oncol Rep.

[23] Mehanna H, Wong WL, McConkey CC, Rahman JK, Robinson M, Hartley AG (2016). PET-CT surveillance versus neck dissection in advanced head and neck cancer. N Engl J Med.

[24] Moeller BJ, Rana V, Cannon BA, Williams MD, Sturgis EM, Ginsberg LE (2009). Prospective risk-adjusted [18F]Fluorodeoxyglucose positron emission tomography and computed tomography assessment of radiation response in head and neck cancer. J Clin Oncol.

[25] Semrau S, Haderlein M, Schmidt D, Lell M, Wolf W, Waldfahrer F (2015). Single-cycle induction chemotherapy followed by chemoradiotherapy or surgery in patients with head and neck cancer: what are the best predictors of remission and prognosis?. Cancer.

[26] Kikuchi M, Shinohara S, Nakamoto Y, Usami Y, Fujiwara K, Adachi T (2011). Sequential FDG-PET/CT after neoadjuvant chemotherapy is a predictor of histopathologic response in patients with head and neck squamous cell carcinoma. Mol Imaging Biol.

[27] Weber WA, Wieder H (2006). Monitoring chemotherapy and radiotherapy of solid tumors. Eur J Nucl Med Mol Imaging.

[28] Schoder H, Fury M, Lee N, Kraus D (2009). PET monitoring of therapy response in head and neck squamous cell carcinoma. J Nucl Med.

[29] Brun E, Kjellen E, Tennvall J, Ohlsson T, Sandell A, Perfekt R (2002). FDG PET studies during treatment: prediction of therapy outcome in head and neck squamous cell carcinoma. Head Neck.

