# Supplementary material for: The role of interim FDG PET-CT after induction chemotherapy as a predictor of concurrent chemoradiotherapy efficacy and prognosis for head and neck cancer
Source: Eur J Nucl Med Mol Imaging. 2017 Sep 22;45(2):170–8. doi: 10.1007/s00259-017-3836-8 (PMC5745569; doi:10.1007/s00259-017-3836-8)
Supplement: Supplementary file 1 — (DOC 92 kb) [file 259_2017_3836_MOESM1_ESM.doc]

**Supplementary Table 1. LN SUVmax and MTV versus CR, PFS, and OS**

|  | **Cutoff** | **Below cutoff (n)** | **Above cutoff (n)** | ***p*-value for CR** | ***p*-value for PFS** | ***p*-value for OS** |
| --- | --- | --- | --- | --- | --- | --- |
| **LN SUVmax decrease (%)** | 62% | 14 | 29 | 0.091 | 0.022 | 0.007 |
|  | 64% | 15 | 28 | 0.029 | 0.007 | 0.002 |
|  | 65% | 17 | 26 | 0.103 | 0.039 | 0.008 |
|  | 71% | 19 | 24 | 0.029 | 0.021 | 0.010 |
|  | 73%* | 20 | 23 | 0.007 | 0.011 | 0.003 |
|  | 75% | 21 | 22 | 0.016 | 0.022 | 0.006 |
|  | 76% | 22 | 21 | 0.031 | 0.035 | 0.012 |
|  | 77% | 23 | 20 | 0.014 | 0.014 | 0.021 |
|  | 78% | 24 | 19 | 0.017 | 0.036 | 0.034 |
|  | 80% | 25 | 18 | 0.007 | 0.056 | 0.047 |
|  | 87% | 26 | 17 | 0.013 | 0.094 | 0.071 |
|  | 89% | 27 | 16 | 0.026 | 0.137 | 0.100 |
|  | 100% | 28 | 15 | 0.051 | 0.071 | 0.037 |
| **MTV decrease (%)** | 68% | 6 | 37 | 0.097 | <0.001 | <0.001 |
|  | 76% | 7 | 36 | 0.042 | <0.001 | <0.001 |
|  | 78%* | 8 | 35 | 0.021 | <0.001 | <0.001 |
|  | 81% | 9 | 34 | 0.061 | <0.001 | <0.001 |
|  | 83% | 10 | 33 | 0.079 | 0.040 | <0.001 |
|  | 87% | 11 | 32 | 0.148 | 0.001 | <0.001 |
|  | 89% | 12 | 31 | 0.084 | 0.002 | <0.001 |
|  | 93% | 14 | 29 | 0.022 | 0.001 | <0.001 |
|  | 94% | 17 | 26 | 0.030 | <0.001 | <0.001 |
|  | 96% | 19 | 24 | 0.105 | <0.001 | <0.001 |
|  | 97% | 20 | 23 | 0.034 | <0.001 | <0.001 |
|  | 98% | 22 | 21 | 0.031 | <0.001 | <0.001 |
|  | 99% | 25 | 18 | 0.032 | <0.001 | <0.001 |
|  | 100% | 27 | 16 | 0.026 | 0.002 | 0.003 |

Abbreviations: LN, lymph node; SUVmax, maximum standardized uptake value; MTV, metabolic tumor volume; CR, complete response; PFS, progression-free survival; OS, overall survival

* Selected for further evaluation.

**Supplementary Table 2. Univariate and multivariate analyses of complete response to concurrent chemoradiotherapy, progression-free survival and overall survival in subgroups.**

1. Nasopharynx group (N=15) : no significant.
2. Non-nasopharynx group (N=28).

| **Variables** | **HR** | **95% CI** | ***p*-value** |
| --- | --- | --- | --- |
| **CR to CCRT** |  |  |  |
| **Univariate analysis** |  |  |  |
| SUVmax of LN decrease ≥ 73% | 6.6 | 1.2-35.7 | 0.014 |
| Total MTV decrease ≥ 78% | 7.0 | 1.1–45.5 | 0.021 |
| **Multivariate analysis** |  |  |  |
| SUVmax of LN decrease ≥ 73% | 6.6 | 1.2-35.7 | 0.014 |
| **PFS** |  |  |  |
| **Univariate analysis** |  |  |  |
| Performance status 0-1 | 2.4 | 1.2-4.7 | 0.005 |
| SUVmax of LN decrease ≥ 73% | 3.4 | 1.1-11.1 | 0.019 |
| Total MTV decrease ≥ 78% | 6.2 | 2.0–19.2 | 0.001 |
| **Multivariate analysis** |  |  |  |
| Total MTV decrease ≥ 78% | 6.2 | 2.0-19.2 | 0.001 |
| **OS** |  |  |  |
| **Univariate analysis** |  |  |  |
| Performance status 0-1 | 3.1 | 1.5-6.4 | 0.001 |
| SUVmax of LN decrease ≥ 73% | 4.3 | 1.2-15.6 | 0.014 |
| Total MTV decrease ≥ 78% | 9.6 | 2.9-32.3 | <0.001 |
| **Multivariate analysis** |  |  |  |
| Total MTV decrease ≥ 78% | 9.6 | 2.9-32.3 | <0.001 |

Abbreviations: LN, lymph node; SUVmax, maximum standardized uptake value; MTV, metabolic tumor volume; CR, complete response; CCRT, Concurrent chemoradiotherapy; PFS, progression-free survival; OS, overall survival
